# The Effect of Atopy in the Prevalence of Contact Sensitization: The Experience of a Greek Referral Center

**DOI:** 10.1155/2020/3946084

**Published:** 2020-10-09

**Authors:** Anna Tagka, George I. Lambrou, Electra Nicolaidou, Stamatios G. Gregoriou, Alexandra Katsarou-Katsari, Dimitrios Rigopoulos

**Affiliations:** ^1^First Department of Dermatology and Venereology, “Andreas Syggros” Hospital, National and Kapodistrian University of Athens, Medical School, Ionos Dragoumi 5, Athens 11621, Greece; ^2^First Department of Pediatrics, Choremeio Research Laboratory, National and Kapodistrian University of Athens, Thivon & Levadeias 8 Goudi, Athens 11527, Greece

## Abstract

Contact dermatitis is a well-known skin condition, which is related to stimuli and environmental exposure to chemicals, affecting all ages as well as both genders. In the present work, we attempt to investigate the patterns of contact sensitization, with respect to the personal history of atopy (AT), in Greece in a large number of allergens, using patch testing. The retrospective analysis included clinical routine data of 1978 patients collected from 2014 to 2016 in the Laboratory of Patch Testing, National Referral Centre of Occupational Dermatoses. Sensitization, in all cases, was tested with 28 allergens of the European baseline series as adjusted to our local circumstances and clinical experience. A total population of 1978 patients was evaluated, with a male-to-female ratio of 0.45 (1359 females/619 males). From our patient cohort, 693 (35%) patients were evaluated with a history of atopy, while 1285 (65%) were nonatopic. The five most prevalent allergens in the total population without AT were nickel sulphate 5% (15.47%), fragrance mix (I) 8% (9.10%), balsam of Peru (6.47%), cobalt chloride 1% (4.70%), and thiomersal 0.1% (4.10%). Respectively, in the total population with AT, the five most prevalent allergens were nickel sulphate 5% (10.36%), fragrance mix (I) 8% (5.11%), balsam of Peru (3.29%), thiomersal 0.1% (3.03%), and cobalt chloride 1% (2.78%). Contact dermatitis surveillance is of great importance towards the clinical and systematic understanding of the disease. Further studies should be directed towards that end, in order to facilitate more effective health policies.

## 1. Introduction

Contact dermatitis is a common skin disorder related to environmental exposures affecting all age groups as well as both genders. According to *Gell & Coombs* classification, “*the type IV reaction of delayed-type hypersensitivity is evaluated, in patients previously sensitized, which present clinical signs of contact dermatitis. Patch testing is a simple in vivo well-established method to diagnose allergic contact dermatitis*” [1]. This method (patch testing) consists of a very practical test, which facilitates the evaluation and diagnosis of allergic contact dermatitis through the skin exposure to the responsible allergens.

European baseline series includes several categories of metals, fragrances, preservatives, rubbers, topical therapeutics, and excipients, which are all considered to be allergenic factors causing eventually contact dermatitis [2]. Data collection from the European Surveillance System on Contact Allergies (ESSCA Network) has a task to assess the structure of patients' sample (between countries and departments) and the overall yield concerning to baseline series. The pattern of contact sensitization to a series of allergens included in the European baseline series has already been studied for a number of EU countries by the ESSCA Network [3–12]. Patch testing, employing allergens, is a method of choice for the detection of sensitization to allergens. Yet, patch test results often vary between departments and laboratories, as well as between countries. Such variations may be partly attributable to systematic effects introduced by patient characteristics, differing exposures, patient selection, or methodological differences. Recommendations of the European Society of Contact Dermatitis (ESCD) are regarded through appropriate changes for specific conditions of exposure in Greece according to labor, commercial, social, and national habits [2, 13].

On the other hand, *atopy* (AT) is a “*predisposition toward developing certain allergic hypersensitivity reactions*” [14, 15]. Atopy may have a genetic predisposition, although contact with an allergen or irritant may be a necessity, before a hypersensitivity reaction can develop [14, 15]. The term *atopy* was first proposed by *Coca* & *Cooke* in 1923 [16]. In general, the term “*atopy*” is used for any immunological reaction that is mediated through IgE reaction, while in other cases it is referred only to the cases that are believed to have a genetic predisposition. The term “*atopy*” is derived from the Greek word *“$\overset{'}{\alpha }$τοπíα*”, which means “*the state of being out of place*”, “*absurdity*”. A form of atopy is *atopic dermatitis*, which is also known as *atopic eczema*. Atopic dermatitis is considered to be an inflammation of the skin, resulting in an itchy, swollen, and cracked skin [17, 18]. Atopic dermatitis is mainly a disease of childhood, whereas almost 20% of children will be presented with symptoms of the disease during their lives and 95% of those manifesting symptoms are younger than five years of age [19]. The majority overcomes atopic dermatitis in adolescence; however, 25% continue to present hand eczema in adulthood [19]. In addition, it has been reported that the disease reoccurs in the elderly with a concurrent severity increase with age [20, 21].

The current work aims to explore the patterns of sensitization to contact allergens, through patch testing, against a large number of allergens provoking contact dermatitis, in Greece. Contact allergens included the European baseline series as adjusted to the local experience. Contact sensitization prevalence is investigated with respect to the presence or not of previous personal history of atopy. Thus, we have investigated the positive reactions of contact allergens in patients with atopy or not. Towards that purpose, we applied the baseline series and available additional series according to the medical history of the patient [22, 23]. To the best of our knowledge, this is the first report concerning the study of atopy-related contact sensitization in Greece.

## 2. Materials and Methods

### 2.1. Patients

A total number of 1978 Greek Caucasian patients (619M/1359F) were admitted in our laboratory during the period between 2014 and 2016 (total of three years). Patients were recruited based on their dermatological profile, whereas all other biometric and anthropometric criteria were kept random. The mean age of all patients was 45.91 ± 18.60 years, where males were 47.31 ± 20.56 years old and females were 45.27 ± 17.60 years old. The reported results refer to consecutive patients in order to avoid bias due to selective testing. Sensitization in all cases was tested with a battery of 28 allergens according to the European baseline series (with addition or omission of allergens due to individual country circumstances) and additional series aiming to identify sensitizations in order to inform the national baseline of allergens. The results refer to consecutive patients in order to avoid bias due to selective testing. Sensitization in all cases was tested with a battery of 28 allergens according to the European baseline series (with addition or omission of allergens due to individual country circumstances) and additional series aiming to identify sensitizations in order to inform the national baseline of allergens. The results refer to consecutive patients in order to avoid bias due to selective testing. Sensitization in all cases was tested with a battery of 28 allergens according to the European baseline series (with addition or omission of allergens due to individual country circumstances) and additional series aiming to identify sensitizations in order to inform the national baseline of allergens.

### 2.2. Inclusion Criteria

All patients admitted to our department, with the suspicion of contact dermatitis, were included in the present study [24, 25]. Patients were selected randomly, in order to avoid biased selective inclusion.

### 2.3. Exclusion Criteria

Patients admitted who were under some kind of anti-inflammatory treatment, under cyclosporine treatment, chronically use corticosteroids, under chemotherapeutics treatments as well as suffering from other chronic dermatopathies were excluded from the present study. Although there is no consensus for the definitive criteria of exclusion from a patch testing procedure, most studies agree that the use of corticosteroids [26], immunodepressants (such as cyclosporine and chemotherapeutics) [27–30], anti-inflammatory treatments [22, 31], other chronic dermatopathies [26], and high exposure to UV [32] (as for example during the summer) are factors that might produce false-negative or false-positive results and thus should be avoided.

### 2.4. Patient Stratification

Patients have been stratified according to the following criteria: (a) the personal history or not of an atopy, where all patients who reported a personal history of any of the following such as allergic rhinitis, asthma, conjunctivitis, atopic dermatitis were sorted as patients with a personal history of atopy, and (b) the gender and (c) the profession, based on the International Standard Classification of Occupations (ISCO). Patients were separated into “blue collars” (BlC) indicating those that performed a handicraft and “white collars” (WhC) indicating those working in an office environment or performing mostly an intellectual profession.

### 2.5. Patch Testing and Clinical Evaluation

The diagnosis of contact dermatitis included the detection of at least one positive reaction to the chemicals panel used in the present study. The retrospective analysis included routine data collected in the Laboratory of Patch Testing, National Referral Centre of Occupational Dermatoses, University Hospital “*Andreas Syggros*,” National and Kapodistrian University of Athens, Medical School [10].

We performed the patch testing according to the guidelines of the European Society of Contact Dermatitis [22]. The optimal exposure time was considered to be two days (48 h). Based on the respective guidelines, it is recommended to perform a test reading two times, where the first takes place after the removal of the patches (48 h) and the second 2–4 days later. The maximum reaction was assessed between day 3 and day 4 after the application of allergens, in the majority of patients. The third reading at day7 was recommended, in order to reveal positive reactions to either slow-reacting allergens (neomycin and corticosteroids) which are late reactors. The reaction was assessed as positive/allergic in terms of morphology lesions on a scale of (i) *weak +*, (ii) *strong ++*, and (iii) *extreme +++*, according to the criteria of the International Contact Dermatitis Research Group (ICDRG) and everything else as negative (including irritant reactions). This was assessed as the patch test outcome. Patch was applied at the middle upper back, which is considered the ideal anatomical position for patch testing. Patches were always applied on a hairless and free of lesions skin. In the present study, we reported the sensitization results only, without referring to the relevance of the patients' clinical image and contact allergens.

## 3. Clinical Parameters and Data Collection

### 3.1. Clinical Parameters

The MOAHLFAP index [9] included characteristics of patients such as *M* (male), *O* (occupational dermatitis (OD)), *A* (atopic dermatitis (AD)), *H* (hand dermatitis (HD)), *L* (leg dermatitis (LD)), *F* (face dermatitis (FD)), *A* (age 40+), and *P* (at least one positive). Further on, we have used the criterion of trunk and generalized dermatitis in our patients [8]. This index contributes to the group description, stratifying the results as to the presence of sensitization prevalence and provides a multifunctional analysis in order to estimate the risk of sensitization (for example, being male with occupational contact dermatitis) [33]. The index has been previously described by our team [13].

### 3.2. Clinical Data

The analysis included the collection of routine data from our laboratory performing the patch test. We collected demographic, clinical data, and patch testing results related to patients suspected with contact dermatitis. The results were documented to an electronic database. In case of patient's repeated admittance, during our study period, only the initial patch test result was considered. We calculated the overall prevalence of at least one positive reaction to a hapten of the EBS in the study population, in the different age groups and in patients with and without AT (atopy). AT was evaluated according to the *Hanifin* and *Rajka* criteria [34].

### 3.3. Data Analysis

Patient's characteristics are presented with absolute and relative frequencies (%). The proportion of positive reactors was also calculated. This proportion was further adjusted over age and sex, following the pertinent guidelines for the statistical analysis of patch test data [35, 36], using the *Segi* world standard population distribution [37]. The most common allergens by patient characteristics are also presented with absolute and relative frequencies (%). Data are available upon reasonable request.

### 3.4. Ethics Statement

The protocol of our study was approved by the Institutional Scientific Review Board of the University Hospital “*Andreas Syggros*,” National and Kapodistrian University of Athens, Medical School (Protocol. Nr. 2851/2018), and the ethical considerations were fully consistent with the Declaration of Helsinki (1975, review 2000). The data were kept anonymously, and there is no way to track back to the patient's personal data.

## 4. Results and Discussion

### 4.1. Patient Cohort Descriptive Statistics

Patient descriptive statistics have been extensively evaluated as we have investigated all major as well as minor groups of our cohort. Our patient population sample is summarized in Table 1.

### 4.2. Frequencies of the Most Prevalent Allergens in the Total Population with and without Atopy

#### 4.2.1. Frequencies of the Most Prevalent Allergens in the Total Population without Atopy

From our results, it appeared that in the total population without AT, the five most prevalent allergens were nickel sulphate 5% (15.47%), followed by other allergens in the following descending order: fragrance mix (I) 8% (9.10%), balsam of Peru (6.47%), cobalt chloride 1% (4.70%), and thiomersal 0.1% (4.10%). Respectively, in the male population, the five most prevalent allergens were fragrance mix (I) 8% (2.78%), balsam of Peru (2.73%), nickel sulphate 5% (2.48%), potassium dichromate 0.5% (1.92%), and cobalt chloride 1% (1.77%). Finally, in the female population, the five most prevalent allergens were nickel sulphate 5% (12.99%), fragrance mix (I) 8% (6.32%), balsam of Peru (3.74%), cobalt chloride 1% (2.93%), and thiomersal 0.1% (2.43%). The relative frequencies of the most prevalent allergens are summarized in Table 2.

#### 4.2.2. Frequencies of the Most Prevalent Allergens in the Total Population with Atopy

From our results, it appeared that in the total population with AT, the five most prevalent allergens were nickel sulphate 5% (10.36%), followed by other allergens in the following descending order: fragrance mix (I) 8% (5.11%), balsam of Peru (3.29%), thiomersal 0.1% (3.03%), and cobalt chloride 1% (2.78%). Respectively, in the male population, the five most prevalent allergens were balsam of Peru (1.21%), nickel sulphate 5% (0.96%), fragrance mix (I) 8% (0.86%), thiomersal 0.1% (0.76%), and potassium dichromate 0.5% (0.61%). Finally, in the female population with AT, the five most prevalent allergens were nickel sulphate 5% (9.40%), fragrance mix (I) 8% (4.25%), cobalt chloride 1% (2.33%), thiomersal 0.1% (2.28%), and balsam of Peru (2.07%). The relative frequencies of the most prevalent allergens are summarized in Table 3.

### 4.3. Frequencies of the Most Prevalent Allergens in the Blue-Collar Population with and without Atopy

#### 4.3.1. Frequencies of the Most Prevalent Allergens in the Blue-Collar Population without Atopy

From our results, it appeared that in the blue-collar population without AT, the five most prevalent allergens were nickel sulphate 5% (4.80%), followed by other allergens in the following descending order: fragrance mix (I) 8% (2.63%), balsam of Peru (1.72%), cobalt chloride 1% (1.37%), and paraphenylenediamine 1% (4.10%). Respectively, in the male blue-collar population, the five most prevalent allergens were potassium dichromate 0.5% (0.91%), cobalt chloride 1% (0.81%), nickel sulphate 5% (0.66%), thiomersal 0.1% (0.56%), and balsam of Peru 25% (0.56%), as well as fragrance mix (I) 8% (0.51%) and paraphenylenediamine 1% (0.51%). Finally, in the female blue-collar population, the five most prevalent allergens were nickel sulphate 5% (4.15%), fragrance mix (I) 8% (2.12%), balsam of Peru 25% (1.16%), paraphenylenediamine 1% (0.86%), and cobalt chloride 1% (0.56%). The relative frequencies of the most prevalent allergens in the AT-negative blue-collar population are summarized in Table 4.

#### 4.3.2. Frequencies of the Most Prevalent Allergens in the Blue-Collar Population with Atopy

From our results, it appeared that in the blue-collar population with AT, the five most prevalent allergens were nickel sulphate 5% (2.53%), followed by other allergens in the following descending order: fragrance mix (I) 8% (1.37%), potassium dichromate 0.5% (0.86%), cobalt chloride 1% (0.71%), and balsam of Peru 25% (0.71%) as well as paraphenylenediamine 1% (0.66%). Respectively, in the male blue-collar population, the five most prevalent allergens were nickel sulphate 5% (0.40%), potassium dichromate 0.5% (0.35%), balsam of Peru 25% (0.25%), cobalt chloride 1% (0.20%), and fragrance mix (I) 8% (0.15%). Finally, in the female blue-collar population, the five most prevalent allergens were nickel sulphate 5% (2.12%), fragrance mix (I) 8% (1.21%), paraphenylenediamine 1% (0.61%), potassium dichromate 0.5% (0.51%), and cobalt chloride 1% (0.51%) and ethylenediamine 1% (0.51%), as well as balsam of Peru 25% (0.46%). The relative frequencies of the most prevalent allergens in the AT-positive blue-collar population are summarized in Table 5.

### 4.4. Frequencies of the Most Prevalent Allergens in the White-Collar Population with and without Atopy

#### 4.4.1. Frequencies of the Most Prevalent Allergens in the White-Collar Population without Atopy

Similar to the previous analyses, we have investigated the prevalence of allergens in the white-collar population without AT. From our results, it appeared that in the white-collar population without AT, the five most prevalent allergens were nickel sulphate 5% (10.67%), followed by other allergens in the following descending order: fragrance mix I (8%) (6.47%), balsam of Peru 25% (4.75%), cobalt chloride 1% (3.34%), and thiomersal 0.1% (3.03%). Respectively, in the male white-collar population, the five most prevalent allergens were fragrance mix (I) 8% (2.28%), balsam of Peru 25% (2.17%), nickel sulphate 5% (1.82%), ethylenediamine 1% (1.26%), and thiomersal 0.1% (1.11%). Finally, in the female white-collar population, the five most prevalent allergens were nickel sulphate 5% (8.85%), fragrance mix (I) 8% (4.20%), balsam of Peru 25% (2.58%), cobalt chloride 1% (2.38%), and thiomersal 0.1% (1.92%). The relative frequencies of the most prevalent allergens in the AT-negative white-collar population are summarized in Table 6.

#### 4.4.2. Frequencies of the Most Prevalent Allergens in the White-Collar Population with Atopy

Similar to the previous analyses, we have investigated the prevalence of allergens in the white-collar population with AT. From our results, it appeared that in the white-collar population with AT, the five most prevalent allergens were nickel sulphate 5% (7.84%), followed by other allergens in the following descending order: fragrance mix (I) 8% (3.74%), thiomersal 0.1% (2.68%), balsam of Peru 25% (2.58%), and cobalt chloride 1% (2.07%). Respectively, in the male white-collar population, the five most prevalent allergens were balsam of Peru 25% (0.96%), fragrance mix (I) 8% (0.71%), thiomersal 0.1% (0.66%), nickel sulphate 5% (0.56%), and ethylenediamine 1% (0.40%). Finally, in the female white-collar population, the five most prevalent allergens were nickel sulphate 5% (7.28%), fragrance mix (I) 8% (3.03%), thiomersal 0.1% (2.02%), cobalt chloride 1% (1.82%), and balsam of Peru 25% (1.62%). The relative frequencies of the most prevalent allergens in the AT-positive white-collar population are summarized in Table 7.

### 4.5. Common Allergens between Patients with and without Atopy

**Figure 1:**
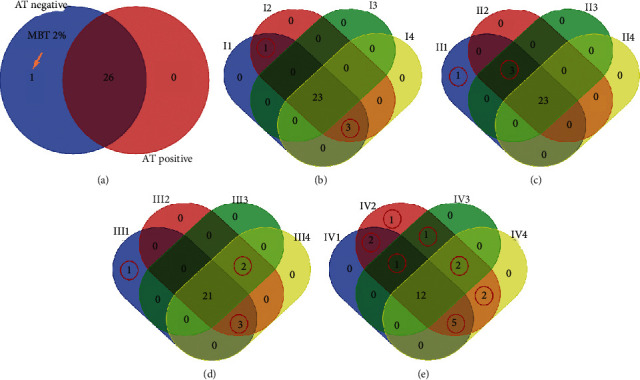
Venn diagrams of AT-negative and AT-positive subpopulations. AT-negative patients were uniquely sensitized by MBT 2% (a). In addition, benzalkonium chloride 0.1%, quinoline mix 6%, and mercapto mix 2% sensitized commonly the AT-negative females, AT-negative males, and AT-positive females, while MBT 2% was common to AT-negative females and AT-negative males (b). Further on, MBT 2% uniquely sensitized AT-negative WhC, while AT-negative BlC, AT-negative WhC, and AT-positive WhC were commonly sensitized by benzalkonium chloride 0.1%, primin 0.01%, and black rubber mix 0.1% (c). The next comparison revealed that AT-negative BlC females, AT-negative WhC females, and AT-negative WhC males were commonly sensitized by primin 0.01%, quinoline mix 6%, and paratertiary butylphenol 1%. At the same time, AT-negative BlC females, AT-negative WhC females, and AT-negative BlC males were commonly sensitized by benzocaine 5% and mercapto mix 2%. Finally, AT-negative WhC males were uniquely sensitized by MBT 2% (d). In the case of AT-positive patients with respect to gender and occupation, more common and unique allergens were found. In particular, finally, epoxy resin 1% uniquely sensitized the AT-positive WhC females, AT-positive BlC males, and AT-positive WhC males. Further on, wool alcohols 30% uniquely sensitized AT-positive WhC females and AT-positive BlC males, while benzalkonium chloride 0.1% uniquely sensitized the AT-positive WhC males. In addition, AT-positive BlC females, AT-positive WhC females, and AT-positive BlC males were commonly sensitized by mercury 0.05% and 5-chloro-2-methyl-4-itz-3. Similarly, AT-positive WhC females and AT-positive WhC males were commonly sensitized by primin 0.01% and black rubber mix 0.1%. AT-positive BlC females and AT-positive WhC females were commonly sensitized by quinoline mix 6% and mercapto mix 2%. Finally, AT-positive BlC females, AT-positive WhC females, and AT-positive WhC males were commonly sensitized by quaternium-15 1%, paraben mix 15%, neomycin sulphate 20%, paratertiary butylphenol 1%, and colophony 20% (e). **AT**: atopy, WhC: white collars, BlC: blue collars, I1: AT-negative males, I2: AT-negative females, I3: AT-positive males, I4: AT-positive females. II1: AT-negative white collars, II2: AT-negative blue collars, II3: AT-positive white collars, II4: AT-positive blue collars. III1: AT-negative white-collar males, III2: AT-negative white-collar females, III3: AT-negative blue-collar males, III4: AT-negative blue-collars females. IV1: AT-positive white-collar males, IV2: AT-positive white-collar females, IV3: AT-positive blue-collar males, and IV4: AT-positive blue-collar females.

In order to examine further patterns in allergen sensitization with respect to AT, we have analyzed the tested allergens with respect to gender and occupation (WhC and BlC). In particular, we have found that AT-negative patients were uniquely sensitized by MBT 2% (Figure 1(a)). In addition, benzalkonium chloride 0.1%, quinoline mix 6%, and mercapto mix 2% sensitized commonly the AT-negative females, AT-negative males, and AT-positive females, while MBT 2% was common to AT-negative females and AT-negative males **(**Figure 1(b)). Further on, MBT 2% uniquely sensitized AT-negative WhC, while AT-negative BlC, AT-negative WhC, and AT-positive WhC were commonly sensitized by benzalkonium chloride 0.1%, primin 0.01%, and black rubber mix 0.1% (Figure 1(c)). The next comparison revealed that AT-negative BlC females, AT-negative WhC females, and AT-negative WhC males were commonly sensitized by primin 0.01%, quinoline mix 6%, and paratertiary butylphenol 1%. At the same time, AT-negative BlC females, AT-negative WhC females, and AT-negative BlC males were commonly sensitized by benzocaine 5% and mercapto mix 2%. Finally, AT-negative WhC males were uniquely sensitized by MBT 2% (Figure 1(d)).

In the case of AT-positive patients with respect to gender and occupation, more common and unique allergens were found. In particular, finally, epoxy resin 1% uniquely sensitized the AT-positive WhC females, AT-positive BlC males, and AT-positive WhC males. Further on, wool alcohols 30% uniquely sensitized AT-positive WhC females and AT-positive BlC males, while benzalkonium chloride 0.1% uniquely sensitized the AT-positive WhC males. In addition, AT-positive BlC females, AT-positive WhC females, and AT-positive BlC males were commonly sensitized by mercury 0.05% and 5-chloro-2-methyl-4-itz-3. Similarly, AT-positive WhC females and AT-positive WhC males were commonly sensitized by primin 0.01% and black rubber mix 0.1%. AT-positive BlC females and AT-positive WhC females were commonly sensitized by quinoline mix 6% and mercapto mix 2%. Finally, AT-positive BlC females, AT-positive WhC females, and AT-positive WhC males were commonly sensitized by quaternium-15 1%, paraben mix 15%, neomycin sulphate 20%, paratertiary butylphenol 1%, and colophony 20% (Figure 1(e)). The results are also summarized in Table 8.

### 4.6. Risk Assessment of Allergens with respect to Occupation

Risk measures with respect to AT manifested several few interesting results. In particular, it appeared that AT did not manifest any significant risk with respect to any allergen, with the exceptions of cocamidopropyl 1% (OR = 0.36, *p*=0.01), *D. pteronyssinus* (OR = 0.16, *p*=2.3 × 10^−7^), and *D. farinae* (OR = 0.19, *p*=1.34 × 10^−5^). Thus, it appeared that patients with AT had a 59% higher risk to be sensitized by cocamidopropyl 1%, 25% higher risk to be sensitized by *D. pteronyssinus*, and 18% higher risk to be sensitized by *D. farinae*. Results are also summarized in Table 9.

## 5. Discussion

The most prevalent allergens in AT patients were found to be nickel sulphate, fragrance mix (I) 8%, balsam of Peru, thiomersal, and cobalt chloride. This finding was in agreement with previous studies which reported that nickel sulphate was also the most prevalent allergen in patient cohorts [38–40]. Similar results have been also previously reported for fragrance mix [41–43], balsam of Peru [44–47], thiomersal [48], and cobalt chloride [44, 49, 50]. The relation of atopies and atopic dermatitis/atopies to allergen sensitization has been discussed in the literature mostly for pediatric populations [51, 52], yet very few reports [53, 54] are available for adult populations, which is a question our present work addresses. Although there is a close relation between the presence of atopy and contact dermatitis, not much is known about the effect of atopy in the allergen contact sensitization. Recent reports have shown that there is a difference in allergen sensitization with respect to the healthy and diseased skin as well as with respect to chronic dermatitis and atopic dermatitis [55, 56]. Moreover, in a recent report, it has been shown that skin inflammations make the skin more prone to sensitization to less potent allergens [57]. In another recent report, it has been shown that several molecular mechanisms are involved in the atopy-induced contact sensitization, such as the compromised chelation of the metals in the stratum corneum of patients with atopic dermatitis leading to increased metal sensitization [58, 59]. Our results are in agreement with those reported in other European countries, which is an interesting indication of similar lifestyles across Europe. This also indicates that the sources of contact sensitization could be similar across Europe, with a probable tendency of converging lifestyles among its population.

The present study has attempted to report and analyze the effects of atopy, in the prevalence of contact sensitization in a Greek patient cohort. An interesting finding was that AT-negative patients were uniquely sensitized by MBT 2% (2-mercaptobenzothiazole). The relation of contact dermatitis and MBT 2% is well documented [60–62]; however, it is the first time that such a finding is reported. This finding was also confirmed by the fact that MBT 2% uniquely sensitized both genders. Further on, we have investigated the effect of profession separating patients into two categories, i.e., labor workers (blue collars (BlC)) and nonlabor workers (white collars (WhC)). Even in that case, AT-negative patients were uniquely sensitized by MBT 2%. This was an interesting finding, since MBT 2% is found in rubber-containing products and it appeared that the presence of AT was not a risk factor for sensitization to it. A possible explanation could be that patients with AT are cautious as far as their contact with environmental stimuli is concerned, while patients with no AT presence are more prone to come in contact with allergenic chemicals such as MBT 2%.

Further on, AT-negative BlC, AT-negative WhC, and AT-positive WhC were sensitized by benzalkonium chloride 0.1%, primin 0.01%, and black rubber mix 0.1%. Our finding is in agreement with previous reports, which have highlighted that benzalkonium chloride 0.1% is prevalent in patients with occupational and atopic dermatitis [63]. The roles of primin [64–66] and black rubber mix [67, 68] have been also documented in contact dermatitis, yet there are no reports for its connection to atopies. Black rubber mix is closely related to MBT 2%, where its unique presence as a sensitization allergen in AT-negative patients confirms the previous observation on MBT 2% sensitization pattern. Yet, no obvious explanation is available for our observation.

Interestingly, these results are in agreement with recent reports, which suggested that the T-helper cell bias characterizing the immune response to atopic dermatitis lowers the risk for contact sensitization as compared to patients with no atopies [58]. Further on, it has been suggested that patients with AT manifest a skin hyporesponsiveness as compared to non-AT skin, probably due to the elevated levels of T-helper 2 and 17 cells [69, 70].

On the other hand, AT-positive patients, both females and males, were uniquely sensitized by epoxy resin 1%. The role of epoxy resin in contact dermatitis is well documented [62, 71–73]. Interestingly, several reports have shown that BlC patients are sensitized to epoxy resin 1%, while it has been shown that patients with atopy are also uniquely to epoxy resin [74]. Yet, it has been reported that epoxy resin is mostly connected to occupational dermatitis rather than atopy [75–79]. Epoxy resin is found in lacquers used in the cosmetic as well as the furnishing industries. Our finding is in agreement with the epoxy resins' source and also suggests, as expected, that AT is a factor for sensitization to this allergen.

Similarly, AT-positive males and females as well as BlC and WhC were found to have a common allergen which was wool alcohols. Interestingly, several reports have shown that sensitization to wool alcohols occurs in children [80–83]. Our finding is in agreement with a previous report manifesting patients with atopic dermatitis to have a high prevalence of concomitant allergic contact dermatitis including wool alcohols [84].

Also, interestingly we have found that AT-positive WhC males and females were uniquely sensitized by primin 0.01% and black rubber mix 0.1%. Since primin is present in cosmetics, our finding is in agreement with the affected groups. Another possible explanation would be the fact that in recent years, there is a turn of the population to the use of organic products, which use primin as an antibacterial/conservative agent. In addition, black rubber mix is used to make all household rubbers, gloves, shoes, and even synthetic clothing and it is possible that AT is connected to the unique sensitization in the aforementioned group.

Additionally, AT-positive BlC males and WhC females were uniquely sensitized by wool alcohols. This was an interesting finding suggesting that this allergen is probably gender-specific. In the same manner, we have found that AT-positive BlC females and WhC males were uniquely sensitized by quinoline mix 6% and mercapto mix 2%. Those allergens are probably gender-specific as they are present in both genders with AT and in both occupational combinations (that is WhC or BlC females as well as WhC or BlC males).

## 6. Study Limitations

One of the study limitations is the possible first-stage selection bias, which cannot be ruled out. One further difficulty is the comparison of prevalence between countries as well as the inherent differences between the similar departments among different countries.

## 7. Future Perspectives

The topic of contact dermatitis and sensitization is very complex and there is still much more to be learned. A very interesting approach would be to investigate the time course of sensitization since changes in sensitization to allergens is tightly connected to environmental changes and the exposure of humans to them. It is possible that several allergens are extinct or newly manifested due to such alterations such as new health and environmental regulations but also to new habits, respectively.

## 8. Conclusions

The present study showed that the prevalence of contact sensitization in the general population was high, mostly for nickel. Interestingly, patients with the previous history of atopy manifested lower prevalence of sensitization to nickel sulphate, fragrance mix, balsam of Peru, cobalt chloride, and thiomersal as compared to patients with no previous history of atopy. The ongoing high prevalence of nickel sensitization shows the importance of complying with regulations, which include consumer products. Fragrance mix appeared to be the second most prevalent allergen, also suggesting a possible need for change in health policies. The etiology and mechanisms of contact dermatitis are still under investigation. Patients with atopy should be under investigation and sensitization in case that allergic contact dermatitis is suspected. It is estimated that contact dermatitis is influenced by environmental as well as genetic factors and it is still a subject of intensive research. Epidemiological studies are considered of crucial importance towards the understanding of contact sensitization and atopy in the establishment of effective clinical and laboratory tests.

## Figures and Tables

**Table 1:** Patient population and age.

Population					
		Males (*n*)	Females (*n*)	Sum	*p* value^¥^
2014		211	457	668	
2015		201	453	654	
2016		207	449	656	
18 < A < 40		178	521	699	
A > 40		380	773	1153	
A < 18		61	65	126	
White collars		463	900	1363	
Blue collars		156	459	615	
2014	18 < A < 40	56	190	246	
	A > 40	126	246	372	
	A < 18	29	21	50	
2015	18 < A < 40	48	181	229	
	A > 40	133	252	385	
	A < 18	20	20	40	
2016	18 < A < 40	74	150	224	
	A > 40	121	275	396	
	A < 18	12	24	36	
2014	White collars	156	320	476	
	Blue collars	55	137	192	
2015	White collars	157	294	451	
	Blue collars	44	159	203	
2016	White collars	150	286	436	
	Blue collars	57	163	220	

Age (years) (mean ± std. deviation)					
		Males	Females	Both genders	*p* value^¥^

	2014	45.49 ± 21.17	44.66 ± 17.85	44.92 ± 18.95	0.59
	2015	49.35 ± 20.56	44.53 ± 17.26	46.01 ± 18.46	0.002
	2016	47.18 ± 19.85	46.64 ± 17.67	46.81 ± 18.37	0.73
	All years	47.31 ± 20.56	45.27 ± 17.61	45.91 ± 18.60	0.02
	18 < A < 40	30.42 ± 5.92	30.65 ± 6.05	30.60 ± 6.02	0.65
	A > 40	61.08 ± 11.45	57.88 ± 11.23	58.93 ± 11.39	0.0006
	A < 18	10.73 ± 4.09	12.54 ± 4.12	11.67 ± 4.20	0.015
White collars		48.21 ± 22.14	44.01 ± 18.12	45.44 ± 19.67	0.0001
Blue collars		44.60 ± 14.67	47.74 ± 16.30	46.95 ± 15.95	0.033
2014	18 < A < 40	30.52 ± 5.57	30.44 ± 5.92	30.45 ± 5.83	0.93
	A > 40	60.33 ± 10.96	58.47 ± 11.02	59.10 ± 11.02	0.12
	A < 18	9.95 ± 4.05	11.43 ± 4.52	10.57 ± 4.28	0.23
2015	18 < A < 40	30.66 ± 6.05	30.60 ± 6.11	30.62 ± 6.08	0.92
	A > 40	61.77 ± 11.24	57.03 ± 11.54	58.67 ± 11.65	0.008
	A < 18	11.57 ± 4.34	13.15 ± 4.12	12.36 ± 4.25	0.24
2016	18 < A < 40	30.19 ± 6.16	30.99 ± 6.16	30.72 ± 6.17	0.36
	A > 40	61.13 ± 12.18	58.11 ± 11.10	59.03 ± 11.51	0.015
	A < 18	11.25 ± 3.69	13.00 ± 3.72	12.42 ± 3.75	0.19
2014	White collars	30.52 ± 5.57	30.44 ± 5.92	30.45 ± 5.83	0.93
	Blue collars	60.33 ± 10.96	58.47 ± 11.02	59.10 ± 11.02	0.12
2015	White collars	30.66 ± 6.05	30.60 ± 6.11	30.62 ± 6.08	0.92
	Blue collars	61.77 ± 11.24	57.03 ± 11.54	58.67 ± 11.65	0.008
2016	White collars	30.19 ± 6.16	30.99 ± 6.16	30.72 ± 6.17	0.36
	Blue collars	61.13 ± 12.18	58.11 ± 11.10	59.03 ± 11.51	0.015

^*¥*^ *p* value indicates differences between male and female population.

**Table 2:** Frequencies of the most prevalent allergens in the population without atopic dermatitis (AT), with respect to the total population, as well as the male and female population.

AT negative (*n* = 1294)	AT negative (*n* = 1294)			AT-negative males (*n* = 438)			AT-negative females (*n* = 846)		
	Absolute frequency	*f*(%) total population	*f*(%) AT negative	Absolute frequency	*f*(%) total population	*f*(%) AT-negative males	Absolute frequency	*f*(%) total population	*f*(%) AT-negative females
Nickel sulphate 5%	306	15.47	23.65	49	2.48	11.19	257	12.99	30.38
Fragrance mix (I) 8%	180	9.10	13.91	55	2.78	12.56	125	6.32	14.78
Balsam of Peru 25%	128	6.47	9.89	54	2.73	12.33	74	3.74	8.75
Cobalt chloride 1%	93	4.70	7.19	35	1.77	7.99	58	2.93	6.86
Thiomersal 0.1%	81	4.10	6.26	33	1.67	7.53	48	2.43	5.67
Potassium dichromate 0.5%	68	3.44	5.26	38	1.92	8.68	30	1.52	3.55
Ethylenediamine 1%	63	3.19	4.87	34	1.72	7.76	29	1.47	3.43
Paraphenylenediamine 1%	61	3.08	4.71	14	0.71	3.20	47	2.38	5.56
Budesonide 0.01%	38	1.92	2.94	18	0.91	4.11	20	1.01	2.36
5-Chloro-2-methyl-4-itz-3	38	1.92	2.94	11	0.56	2.51	27	1.37	3.19
Formaldehyde 2%	38	1.92	2.94	15	0.76	3.42	23	1.16	2.72
Neomycin sulphate 20%	37	1.87	2.86	8	0.40	1.83	29	1.47	3.43
Thiuram mix 1%	31	1.57	2.40	14	0.71	3.20	17	0.86	2.01
Colophony 20%	29	1.47	2.24	14	0.71	3.20	15	0.76	1.77
Wool alcohols 30%	27	1.37	2.09	12	0.61	2.74	15	0.76	1.77
Black rubber mix 0.1%	18	0.91	1.39	4	0.20	0.91	14	0.71	1.65
Paratertiary butylphenol 1%	17	0.86	1.31	5	0.25	1.14	12	0.61	1.42
Quaternium-15 1%	14	0.71	1.08	3	0.15	0.68	11	0.56	1.30
Paraben mix 15%	13	0.66	1.00	5	0.25	1.14	8	0.40	0.95
Benzocaine 5%	12	0.61	0.93	4	0.20	0.91	8	0.40	0.95
Primin 0.01%	10	0.51	0.77	3	0.15	0.68	7	0.35	0.83
Mercapto mix 2%	9	0.46	0.70	3	0.15	0.68	6	0.30	0.71
Mercury 0.05%	8	0.40	0.62	3	0.15	0.68	5	0.25	0.59
Epoxy resin 1%	8	0.40	0.62	4	0.20	0.91	4	0.20	0.47
Quinoline mix 6%	6	0.30	0.46	1	0.05	0.23	5	0.25	0.59
Benzalkonium chloride 0.1%	5	0.25	0.39	2	0.10	0.46	3	0.15	0.35
MBT 2%	4	0.20	0.31	2	0.10	0.46	2	0.10	0.24
Phenylmercuric acetate 0.05%	0	0.00	0.00	0	0.00	0.00	0	0.00	0.00
Hydroquinone	0	0.00	0.00	0	0.00	0.00	0	0.00	0.00
Petrolatum control 100%	0	0.00	0.00	0	0.00	0.00	0	0.00	0.00

**Table 3:** Frequencies of the most prevalent allergens in the population with atopic dermatitis (AT), with respect to the total population, as well as the male and female population.

AT positive (*n* = 692)	AT positive (*n* = 692)			AT-positive males (*n* = 181)			AT-positive females (*n* = 511)		
	Absolute frequency	*f*(%) total population	*f*(%) AT positive	Absolute frequency	*f*(%) total population	*f*(%) AT-positive males	Absolute frequency	*f*(%) total population	*f*(%) AT-positive females
Nickel sulphate 5%	205	10.36	29.62	19	0.96	10.50	186	9.40	36.40
Fragrance mix (I) 8%	101	5.11	14.60	17	0.86	9.39	84	4.25	16.44
Balsam of Peru 25%	65	3.29	9.39	24	1.21	13.26	41	2.07	8.02
Thiomersal 0.1%	60	3.03	8.67	15	0.76	8.29	45	2.28	8.81
Cobalt chloride 1%	55	2.78	7.95	9	0.46	4.97	46	2.33	9.00
Potassium dichromate 0.5%	42	2.12	6.07	12	0.61	6.63	30	1.52	5.87
Ethylenediamine 1%	38	1.92	5.49	11	0.56	6.08	27	1.37	5.28
Paraphenylenediamine 1%	36	1.82	5.20	3	0.15	1.66	33	1.67	6.46
Formaldehyde 2%	20	1.01	2.89	3	0.15	1.66	17	0.86	3.33
Neomycin sulphate 20%	20	1.01	2.89	4	0.20	2.21	16	0.81	3.13
5-Chloro-2-methyl-4-itz-3	18	0.91	2.60	1	0.05	0.55	17	0.86	3.33
Budesonide 0.01%	17	0.86	2.46	6	0.30	3.31	11	0.56	2.15
Thiuram mix 1%	16	0.81	2.31	6	0.30	3.31	10	0.51	1.96
Colophony 20%	12	0.61	1.73	3	0.15	1.66	9	0.46	1.76
Black rubber mix 0.1%	10	0.51	1.45	1	0.05	0.55	9	0.46	1.76
Benzocaine 5%	10	0.51	1.45	2	0.10	1.10	8	0.40	1.57
Paratertiary butylphenol 1%	8	0.40	1.16	2	0.10	1.10	6	0.30	1.17
Paraben mix 15%	8	0.40	1.16	3	0.15	1.66	5	0.25	0.98
Quaternium-15 1%	7	0.35	1.01	1	0.05	0.55	6	0.30	1.17
Mercapto mix 2%	7	0.35	1.01	0	0.00	0.00	7	0.35	1.37
Epoxy resin 1%	5	0.25	0.72	4	0.20	2.21	1	0.05	0.20
Primin 0.01%	4	0.20	0.58	2	0.10	1.10	2	0.10	0.39
Mercury 0.05%	4	0.20	0.58	1	0.05	0.55	3	0.15	0.59
Quinoline mix 6%	4	0.20	0.58	0	0.00	0.00	4	0.20	0.78
Wool alcohols 30%	3	0.15	0.43	1	0.05	0.55	2	0.10	0.39
Benzalkonium chloride 0.1%	2	0.10	0.29	0	0.00	0.00	2	0.10	0.39
Phenylmercuric acetate 0.05%	0	0.00	0.00	0	0.00	0.00	0	0.00	0.00
Hydroquinone	0	0.00	0.00	0	0.00	0.00	0	0.00	0.00
Petrolatum control 100%	0	0.00	0.00	0	0.00	0.00	0	0.00	0.00
MBT 2%	0	0.00	0.00	0	0.00	0.00	0	0.00	0.00

**Table 4:** Frequencies of the most prevalent allergens in the population without atopic dermatitis (AT), with respect to the blue-collar (BlC) population, as well as the male and female BlC population.

AT negative (*n* = 1294)	AT-negative blue collars (*n* = 441)			AT-negative blue-collar males (*n* = 119)			AT-negative blue-collar females (*n* = 322)		
	Absolute frequency	*f*(%) total population	*f*(%) AT negative	Absolute frequency	*f*(%) total population	*f*(%) AT-negative males	Absolute frequency	*f*(%) total population	*f*(%) AT-negative females
Nickel sulphate 5%	95	4.80	21.54	13	0.66	10.92	82	4.15	25.47
Fragrance mix (I) 8%	52	2.63	11.79	10	0.51	8.40	42	2.12	13.04
Balsam of Peru 25%	34	1.72	7.71	11	0.56	9.24	23	1.16	7.14
Cobalt chloride 1%	27	1.37	6.12	16	0.81	13.45	11	0.56	3.42
Paraphenylenediamine 1%	27	1.37	6.12	10	0.51	8.40	17	0.86	5.28
Potassium dichromate 0.5%	27	1.37	6.12	18	0.91	15.13	9	0.46	2.80
Thiomersal 0.1%	21	1.06	4.76	11	0.56	9.24	10	0.51	3.11
Ethylenediamine 1%	18	0.91	4.08	9	0.46	7.56	9	0.46	2.80
Thiuram mix 1%	16	0.81	3.63	8	0.40	6.72	8	0.40	2.48
Budesonide 0.01%	11	0.56	2.49	5	0.25	4.20	6	0.30	1.86
Colophony 20%	10	0.51	2.27	3	0.15	2.52	7	0.35	2.17
5-Chloro-2-methyl-4-itz-3	9	0.46	2.04	4	0.20	3.36	5	0.25	1.55
Neomycin sulphate 20%	9	0.46	2.04	3	0.15	2.52	6	0.30	1.86
Formaldehyde 2%	8	0.40	1.81	3	0.15	2.52	5	0.25	1.55
Wool alcohols 30%	8	0.40	1.81	3	0.15	2.52	5	0.25	1.55
Quaternium-15 1%	6	0.30	1.36	1	0.05	0.84	5	0.25	1.55
Benzocaine 5%	5	0.25	1.13	3	0.15	2.52	2	0.10	0.62
Epoxy resin 1%	5	0.25	1.13	3	0.15	2.52	2	0.10	0.62
Black rubber mix 0.1%	4	0.20	0.91	1	0.05	0.84	3	0.15	0.93
Mercapto mix 2%	4	0.20	0.91	3	0.15	2.52	1	0.05	0.31
Paratertiary butylphenol 1%	3	0.15	0.68	0	0.00	0.00	3	0.15	0.93
Primin 0.01%	3	0.15	0.68	0	0.00	0.00	3	0.15	0.93
Paraben mix 15%	2	0.10	0.45	1	0.05	0.84	1	0.05	0.31
Quinoline mix 6%	2	0.10	0.45	0	0.00	0.00	2	0.10	0.62
Benzalkonium chloride 0.1%	2	0.10	0.45	1	0.05	0.84	1	0.05	0.31
Mercury 0.05%	2	0.10	0.45	1	0.05	0.84	1	0.05	0.31
Phenylmercuric acetate 0.05%	0	0.00	0.00	0	0.00	0.00	0	0.00	0.00
Hydroquinone	0	0.00	0.00	0	0.00	0.00	0	0.00	0.00
Petrolatum control 100%	0	0.00	0.00	0	0.00	0.00	0	0.00	0.00
MBT 2%	0	0.00	0.00	0	0.00	0.00	0	0.00	0.00

**Table 5:** Frequencies of the most prevalent allergens in the population with atopic dermatitis (AT), with respect to the blue-collar (BlC) population, as well as the male and female BlC population.

AT positive (*n* = 692)	AT-positive blue collars (*n* = 174)			AT-positive blue-collar males (*n* = 37)			AT-positive blue-collar females (*n* = 137)		
	Absolute frequency	*f*(%) total population	*f*(%) AT-positive blue collars	Absolute frequency	*f*(%) total population	*f*(%) AT-positive males	Absolute frequency	*f*(%) total population	*f*(%) AT-positive females
Nickel sulphate 5%	50	2.53	28.74	8	0.40	21.62	42	2.12	30.66
Fragrance mix (I) 8%	27	1.37	15.52	3	0.15	8.11	24	1.21	17.52
Potassium dichromate 0.5%	17	0.86	9.77	7	0.35	18.92	10	0.51	7.30
Cobalt chloride 1%	14	0.71	8.05	4	0.20	10.81	10	0.51	7.30
Balsam of Peru 25%	14	0.71	8.05	5	0.25	13.51	9	0.46	6.57
Paraphenylenediamine 1%	13	0.66	7.47	1	0.05	2.70	12	0.61	8.76
Ethylenediamine 1%	13	0.66	7.47	3	0.15	8.11	10	0.51	7.30
Thiuram mix 1%	10	0.51	5.75	5	0.25	13.51	5	0.25	3.65
Thiomersal 0.1%	7	0.35	4.02	2	0.10	5.41	5	0.25	3.65
Formaldehyde 2%	7	0.35	4.02	2	0.10	5.41	5	0.25	3.65
Budesonide 0.01%	6	0.30	3.45	1	0.05	2.70	5	0.25	3.65
5-Chloro-2-methyl-4-itz-3	4	0.20	2.30	1	0.05	2.70	3	0.15	2.19
Neomycin sulphate 20%	4	0.20	2.30	0	0.00	0.00	4	0.20	2.92
Colophony 20%	4	0.20	2.30	0	0.00	0.00	4	0.20	2.92
Benzocaine 5%	4	0.20	2.30	1	0.05	2.70	3	0.15	2.19
Paratertiary butylphenol 1%	3	0.15	1.72	0	0.00	0.00	3	0.15	2.19
Mercury 0.05%	3	0.15	1.72	1	0.05	2.70	2	0.10	1.46
Epoxy resin 1%	3	0.15	1.72	3	0.15	8.11	0	0.00	0.00
Mercapto mix 2%	2	0.10	1.15	0	0.00	0.00	2	0.10	1.46
Quaternium-15 1%	1	0.05	0.57	0	0.00	0.00	1	0.05	0.73
Paraben mix 15%	1	0.05	0.57	0	0.00	0.00	1	0.05	0.73
Quinoline mix 6%	1	0.05	0.57	0	0.00	0.00	1	0.05	0.73
Wool alcohols 30%	1	0.05	0.57	1	0.05	2.70	0	0.00	0.00
Black rubber mix 0.1%	0	0.00	0.00	0	0.00	0.00	0	0.00	0.00
Benzalkonium chloride 0.1%	0	0.00	0.00	0	0.00	0.00	0	0.00	0.00
Primin 0.01%	0	0.00	0.00	0	0.00	0.00	0	0.00	0.00
Phenylmercuric acetate 0.05%	0	0.00	0.00	0	0.00	0.00	0	0.00	0.00
Hydroquinone	0	0.00	0.00	0	0.00	0.00	0	0.00	0.00
Petrolatum control 100%	0	0.00	0.00	0	0.00	0.00	0	0.00	0.00
MBT 2%	0	0.00	0.00	0	0.00	0.00	0	0.00	0.00

**Table 6:** Frequencies of the most prevalent allergens in the population without atopic dermatitis (AT), with respect to the white-collar (WhC) population, as well as the male and female WhC population.

AT negative (*n* = 1294)	AT-negative white collars (*n* = 843)			AT-negative white-collar males (*n* = 319)			AT-negative white-collar females (*n* = 524)		
	Absolute frequency	*f*(%) total population	*f*(%) AT-negative white collars	Absolute frequency	*f*(%) total population	*f*(%) AT-negative males	Absolute frequency	*f*(%) total population	*f*(%) AT-negative females
Nickel sulphate 5%	211	10.67	25.03	36	1.82	11.29	175	8.85	33.40
Fragrance mix (I) 8%	128	6.47	15.18	45	2.28	14.11	83	4.20	15.84
Cobalt chloride 1%	66	3.34	7.83	19	0.96	5.96	47	2.38	8.97
Balsam of Peru 25%	94	4.75	11.15	43	2.17	13.48	51	2.58	9.73
Thiomersal 0.1%	60	3.03	7.12	22	1.11	6.90	38	1.92	7.25
Paraphenylenediamine 1%	34	1.72	4.03	4	0.20	1.25	30	1.52	5.73
Potassium dichromate 0.5%	41	2.07	4.86	20	1.01	6.27	21	1.06	4.01
Ethylenediamine 1%	45	2.28	5.34	25	1.26	7.84	20	1.01	3.82
5-Chloro-2-methyl-4-itz-3	29	1.47	3.44	7	0.35	2.19	22	1.11	4.20
Neomycin sulphate 20%	28	1.42	3.32	5	0.25	1.57	23	1.16	4.39
Formaldehyde 2%	30	1.52	3.56	12	0.61	3.76	18	0.91	3.44
Budesonide 0.01%	27	1.37	3.20	13	0.66	4.08	14	0.71	2.67
Thiuram mix 1%	15	0.76	1.78	6	0.30	1.88	9	0.46	1.72
Wool alcohols 30%	19	0.96	2.25	9	0.46	2.82	10	0.51	1.91
Black rubber mix 0.1%	14	0.71	1.66	3	0.15	0.94	11	0.56	2.10
Colophony 20%	19	0.96	2.25	11	0.56	3.45	8	0.40	1.53
Benzocaine 5%	7	0.35	0.83	1	0.05	0.31	6	0.30	1.15
Paratertiary butylphenol 1%	14	0.71	1.66	5	0.25	1.57	9	0.46	1.72
Paraben mix 15%	11	0.56	1.30	4	0.20	1.25	7	0.35	1.34
Mercapto mix 2%	5	0.25	0.59	0	0.00	0.00	5	0.25	0.95
Quaternium-15 1%	8	0.40	0.95	2	0.10	0.63	6	0.30	1.15
Mercury 0.05%	6	0.30	0.71	2	0.10	0.63	4	0.20	0.76
Epoxy resin 1%	3	0.15	0.36	1	0.05	0.31	2	0.10	0.38
Primin 0.01%	7	0.35	0.83	3	0.15	0.94	4	0.20	0.76
Quinoline mix 6%	4	0.20	0.47	1	0.05	0.31	3	0.15	0.57
Benzalkonium chloride 0.1%	3	0.15	0.36	1	0.05	0.31	2	0.10	0.38
MBT 2%	4	0.20	0.47	2	0.10	0.63	2	0.10	0.38
Phenylmercuric acetate 0.05%	0	0.00	0.00	0	0.00	0.00	0	0.00	0.00
Hydroquinone	0	0.00	0.00	0	0.00	0.00	0	0.00	0.00
Petrolatum control 100%	0	0.00	0.00	0	0.00	0.00	0	0.00	0.00

**Table 7:** Frequencies of the most prevalent allergens in the population with atopic dermatitis (AT), with respect to the white-collar (WhC) population, as well as the male and female WhC population.

AT positive (*n* = 692)	AT-positive white collars (*n* = 518)			AT-positive white-collar males (*n* = 144)			AT-positive white-collar females (*n* = 374)		
	Absolute frequency	*f*(%) total population	*f*(%) AT-positive white collars	Absolute frequency	*f*(%) total population	*f*(%) AT-positive males	Absolute frequency	*f*(%) total population	*f*(%) AT-positive males
Nickel sulphate 5%	155	7.84	29.92	11	0.56	7.64	144	7.28	38.50
Fragrance mix (I) 8%	74	3.74	14.29	14	0.71	9.72	60	3.03	16.04
Thiomersal 0.1%	53	2.68	10.23	13	0.66	9.03	40	2.02	10.70
Balsam of Peru 25%	51	2.58	9.85	19	0.96	13.19	32	1.62	8.56
Cobalt chloride 1%	41	2.07	7.92	5	0.25	3.47	36	1.82	9.63
Potassium dichromate 0.5%	25	1.26	4.83	5	0.25	3.47	20	1.01	5.35
Ethylenediamine 1%	25	1.26	4.83	8	0.40	5.56	17	0.86	4.55
Paraphenylenediamine 1%	23	1.16	4.44	2	0.10	1.39	21	1.06	5.61
Neomycin sulphate 20%	16	0.81	3.09	4	0.20	2.78	12	0.61	3.21
5-Chloro-2-methyl-4-itz-3	14	0.71	2.70	0	0.00	0.00	14	0.71	3.74
Formaldehyde 2%	13	0.66	2.51	1	0.05	0.69	12	0.61	3.21
Budesonide 0.01%	11	0.56	2.12	5	0.25	3.47	6	0.30	1.60
Black rubber mix 0.1%	10	0.51	1.93	1	0.05	0.69	9	0.46	2.41
Colophony 20%	8	0.40	1.54	3	0.15	2.08	5	0.25	1.34
Paraben mix 15%	7	0.35	1.35	3	0.15	2.08	4	0.20	1.07
Quaternium-15 1%	6	0.30	1.16	1	0.05	0.69	5	0.25	1.34
Benzocaine 5%	6	0.30	1.16	1	0.05	0.69	5	0.25	1.34
Thiuram mix 1%	6	0.30	1.16	1	0.05	0.69	5	0.25	1.34
Mercapto mix 2%	5	0.25	0.97	0	0.00	0.00	5	0.25	1.34
Paratertiary butylphenol 1%	5	0.25	0.97	2	0.10	1.39	3	0.15	0.80
Primin 0.01%	4	0.20	0.77	2	0.10	1.39	2	0.10	0.53
Quinoline mix 6%	3	0.15	0.58	0	0.00	0.00	3	0.15	0.80
Benzalkonium chloride 0.1%	2	0.10	0.39	0	0.00	0.00	2	0.10	0.53
Wool alcohols 30%	2	0.10	0.39	0	0.00	0.00	2	0.10	0.53
Epoxy resin 1%	2	0.10	0.39	1	0.05	0.69	1	0.05	0.27
Mercury 0.05%	1	0.05	0.19	0	0.00	0.00	1	0.05	0.27
Phenylmercuric acetate 0.05%	0	0.00	0.00	0	0.00	0.00	0	0.00	0.00
Hydroquinone	0	0.00	0.00	0	0.00	0.00	0	0.00	0.00
Petrolatum control 100%	0	0.00	0.00	0	0.00	0.00	0	0.00	0.00
MBT 2%	0	0.00	0.00	0	0.00	0.00	0	0.00	0.00

**Table 8:** Textual explanation of the Venn diagram presented in Figure 1.

Names	Total	Elements	
AT negative	1	MBT 2%	Figure 1(a)
AT-negative males (I1)	3	Benzalkonium chloride 0.1%, quinoline mix 6%, mercapto mix 2%	Figure 1(b)
AT-negative females (I2)			
AT-positive females (I3)			
AT-negative females (I2)	1	MBT 2%	Figure 1(b)
AT-negative males (I1)			
AT-negative BlC (II3)	3	Benzalkonium chloride 0.1%, primin 0.01%, black rubber mix 0.1%	Figure 1(c)
AT-negative WhC (II1)			
AT-positive WhC (II3)			
AT-negative WhC (II1)	1	MBT 2%	Figure 1(c)
AT-negative BlC females (III4)	3	Primin 0.01%, quinoline mix 6%, paratertiary butylphenol 1%	Figure 1(d)
AT-negative WhC females (III2)			
AT-negative WhC males (III1)			
AT-negative BlC females (III4)	2	Benzocaine 5%, mercapto mix 2%	Figure 1(d)
AT-negative WhC females (III2)			
AT-negative BlC males (III3)			
AT-negative WhC males (III1)	1	MBT 2%	Figure 1(d)
AT-positive WhC females (IV1)	1	Epoxy resin 1%	Figure 1(e)
AT-positive BlC males (IV3)			
AT-positive WhC males (IV1)			
AT-positive BlC females (IV4)	5	Quaternium-15 1%, paraben mix 15%, neomycin sulphate 20%, paratertiary butylphenol 1%, colophony 20%	Figure 1(e)
AT-positive WhC females (IV2)			
AT-positive WhC males (IV1)			
AT-positive BlC females (IV4)	2	Mercury 0.05%, 5-Chloro-2-Methyl-4-ITZ-3	Figure 1(e)
AT-positive WhC females (IV2)			
AT-positive BlC males (IV3)			
AT-positive WhC females (IV2)	2	Primin 0.01%, black rubber mix 0.1%	Figure 1(e)
AT-positive WhC males (IV1)			
AT-positive WhC females (IV2)	1	Wool alcohols 30%	Figure 1(e)
AT-positive BlC males (IV3)			
AT-positive BlC females (IV4)	2	Quinoline mix 6%, mercapto mix 2%	Figure 1(e)
AT-positive WhC females (IV2)			
AT-positive WhC females (IV2)	1	Benzalkonium chloride 0.1%	Figure 1(e)

**Table 9:** Risk assessment for the presence of atopy.

			Relative risk (RR)	Odds ratio (OR)	Absolute risk (AR)	Fisher's test *p* value	Fisher's test OR	Fisher's test lower CI	Fisher's test upper CI
Cocamidopropyl 1%	Positive	Negative	0.412	0.36	−0.11	0.010	0.36	0.16	0.79
AT yes	9	108							
AT no	33	144							
*D. pteronyssinus*	Negative	Positive	0.75	0.16	−0.23	2.29*E* − 07	0.16	0.078	0.33
AT yes	83	34							
AT no	166	11							
*D. farinae*	Negative	Positive	0.82	0.18	−0.17	1.34*E* − 05	0.1875	0.084	0.42
AT yes	91	26							
AT no	168	9							

## Data Availability

The data used to support the findings of the study are available from the corresponding author upon reasonable request.
